# Novel approach to semi‐quantification of tracer accumulation in dopamine transporter scan

**DOI:** 10.1002/acm2.13626

**Published:** 2022-05-10

**Authors:** Yoshinori Ito, Naotoshi Fujita, Kazuhiro Hara, Tomohiro Tada, Shinji Abe, Masahisa Katsuno, Shinji Naganawa, Katsuhiko Kato

**Affiliations:** ^1^ Department of Radiological and Medical Laboratory Sciences Nagoya University Graduate School of Medicine, Higashi‐ku Nagoya Japan; ^2^ Department of Radiological Technology Nagoya University Hospital, Showa‐ku Nagoya Japan; ^3^ Department of Neurology Nagoya University Graduate School of Medicine, Showa‐ku Nagoya Japan; ^4^ Department of Radiology Nagoya University Graduate School of Medicine, Showa‐ku Nagoya Japan; ^5^ Functional Medical Imaging, Biomedical Imaging Sciences, Division of Advanced Information Health Sciences, Department of Integrated Health Sciences Nagoya University Graduate School of Medicine, Higashi‐ku Nagoya Japan

**Keywords:** FP‐CIT, specific binding ratio, quantification, dopamine transporter, SPECT

## Abstract

**Purpose:**

Accurate tracer accumulation evaluation is difficult owing to the partial volume effect (PVE). We proposed a novel semi‐quantitative approach for measuring the accumulation amount by examining the approximate image. Using a striatal phantom, we verified the validity of a newly proposed method to accurately evaluate the tracer accumulations in the caudate and putamen separately. Moreover, we compared the proposed method with the conventional methods.

**Methods:**

The left and right caudate/putamen regions and the whole brain region as background were identified in computed tomography (CT) images obtained by single‐photon emission computed tomography (SPECT)/CT and acquired the positional information of each region. SPECT‐like images were generated by assigning assumed accumulation amounts to each region. The SPECT‐like image, approximated to the actual measured SPECT image, was examined by changing the assumed accumulation amounts assigned to each region. When the generated SPECT‐like image most approximated the actual measured SPECT image, the accumulation amounts assumed were determined as the accumulation amounts in each region. We evaluated the correlation between the count density calculated by the proposed method and the actual count density of the ^123^I solution filled in the phantom. Conventional methods (CT‐guide method, geometric transfer matrix [GTM] method, region‐based voxel‐wise [RBV] method, and Southampton method) were also evaluated. The significance of differences between the correlation coefficients of various methods (except the Southampton method) was evaluated.

**Results:**

The correlation coefficients between the actual count density and the SPECT count densities were 0.997, 0.973, 0.951, 0.950, and 0.996 for the proposed method, CT‐guide method, GTM method, RBV method, and Southampton method, respectively. The correlation of the proposed method was significantly higher than those of the other methods.

**Conclusions:**

The proposed method could calculate accurate accumulation amounts in the caudate and putamen separately, considering the PVE.

## 1 INTRODUCTION

Dopamine transporter (DaT) scintigraphy with ^123^I‐*N*‐ω‐fluoropropyl‐2β‐carboxymethoxy‐3β‐(4‐iodophenyl)nortropane (^123^I‐FP‐CIT) is useful in the diagnosis of nigrostriatal degenerative diseases such as Parkinson's disease (PD) and dementia with Lewy bodies (DLB).^1^, ^2^, ^3^ Semiquantitative analysis is an adjunct to visual interpretation and improves diagnostic performance.^4^, ^5^ However, considering the spatial resolution of single‐photon emission computed tomography (SPECT), it is difficult to accurately evaluate the accumulation amount of the tracer for small structures such as the striatum, which are affected by the partial volume effect (PVE).^6^, ^7^ Many studies have demonstrated the importance of PVE correction (PVC). Various methods have been developed to correct the PVE, especially in the positron emission tomography (PET) field, while some were applied to the SPECT field.^6^, ^8^, ^9^ The PVC methods are roughly classified into two types: volume of interest (VOI)‐based and voxel‐based methods. The widely applied geometric transfer matrix (GTM) method is a VOI‐based PVC method described by Rousset et al.^10^ This method can account for spill‐out between multiple regions and measure mean values for each region. The region‐based voxel‐wise (RBV) method described by Thomas et al.^11^ is a voxel‐wise PVC method that extends the GTM method. However, the guideline indicates that the added value of applying PVC in calculating the caudate, putamen, and background (BG) uptake has not been systematically investigated and, therefore, is not recommended for routine clinical practice.^4^ A different approach for PVC in DaT SPECT is the Southampton method described by Tossici‐Bolt et al.,^12^ widely used and investigated by many studies.^5^, ^13^, ^14^, ^15^ The Southampton method can calculate the specific binding ratio (SBR) considering the PVE by using a large VOI, including the entire striatum. However, this method cannot calculate the SBR for the caudate and putamen. It has been reported that in PD, the ventrolateral region of the substantia nigra is selectively impaired, resulting in severe dopamine loss in the dorsal putamen compared to the caudate.^16^ Hence, accumulation of ^123^I‐FP‐CIT in PD is especially reduced in the dorsal putamen.^1^ Moreover, in DLB, the accumulation of ^123^I‐FP‐CIT is uniformly reduced in the caudate and putamen.^17^, ^18^ Therefore, separately evaluating the accurate accumulation amount in the caudate and putamen is desirable while diagnosing PD.

This study aimed to verify, using striatal phantom, a newly proposed method to evaluate the accumulation amounts in the caudate and putamen accurately. In addition, the proposed method was compared with conventional PVC methods.

## 2 METHODS

### 2.1 SPECT imaging

SPECT data were acquired using Symbia T (Siemens, Erlangen, Germany), equipped with a low‐medium energy general‐purpose (LMEGP) collimator. Ninety projections over 360° orbit with two detectors were acquired on a 128 × 128 matrix (zoom factor, 1.45), giving a pixel size of 3.3 mm and acquisition time of 28 min. The main energy window was 159 keV ± 10%, and two subwindows were set at 8% at both ends of the main window. Images were reconstructed using a three‐dimensional ordered subset expectation maximization method (3D‐OSEM) (iteration, 6; subset, 8) and Gaussian filter full width at half maximum (FWHM) 6 mm with attenuation correction (AC) by computed tomography (CT) and scatter correction (SC) using the triple energy window method.

### 2.2 Phantom data

**FIGURE 1:**
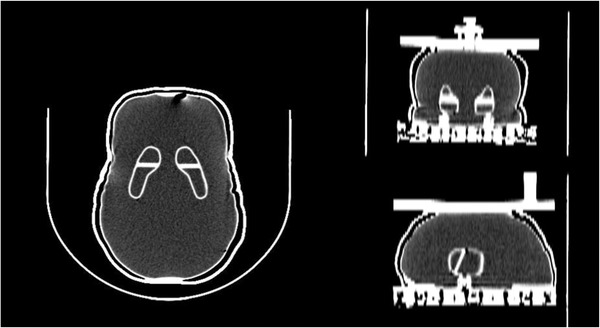
Overview of a striatal phantom. A striatal phantom consists of the left and right caudate/putamen and whole‐brain compartments

Striatal phantom DaT1308 (NMP Business Support Co., Ltd., Hyogo, Japan) was used (Figure 1). This phantom consists of the left and right caudate/putamen and whole‐ brain compartments. We created two types of phantom at different radioactive concentrations of ^123^I solution. In the first phantom (Phantom 1), we filled the left and right caudate with ^123^I solution and the remaining regions with water. We filled all regions with ^123^I solution in the second phantom (Phantom 2). We measured the actual count densities of the ^123^I solution filled into Phantom 1 and Phantom 2 by an auto‐well counter (ARC‐7001, Hitachi, Ltd., Tokyo, Japan) and computed the actual count density ratio. Details of Phantom 1 and Phantom 2 are shown in Table 1.

**TABLE 1:** Phantom data

	**Right caudate (excluding BG)**	**Right putamen (excluding BG)**	**Left caudate (excluding BG)**	**Left putamen (excluding BG)**	**BG**
	Phantom 1				
Actual count density (count/s g)	25123.21	0.00	14156.71	0.00	0.00
Actual ratio	1.77	0.00	1.00	0.00	0.00
	Phantom 2				
Actual count density (count/s g)	21440.22	21440.22	11061.24	8850.12	2337.02
Actual ratio	9.17	9.17	4.73	3.79	1.00

*Note*: Actual count density, the actual ^123^I count density filled in the phantom. Actual ratio, the ratio calculated using by the actual count density.

### 2.3 Calculation process by the proposed method

**FIGURE 2:**
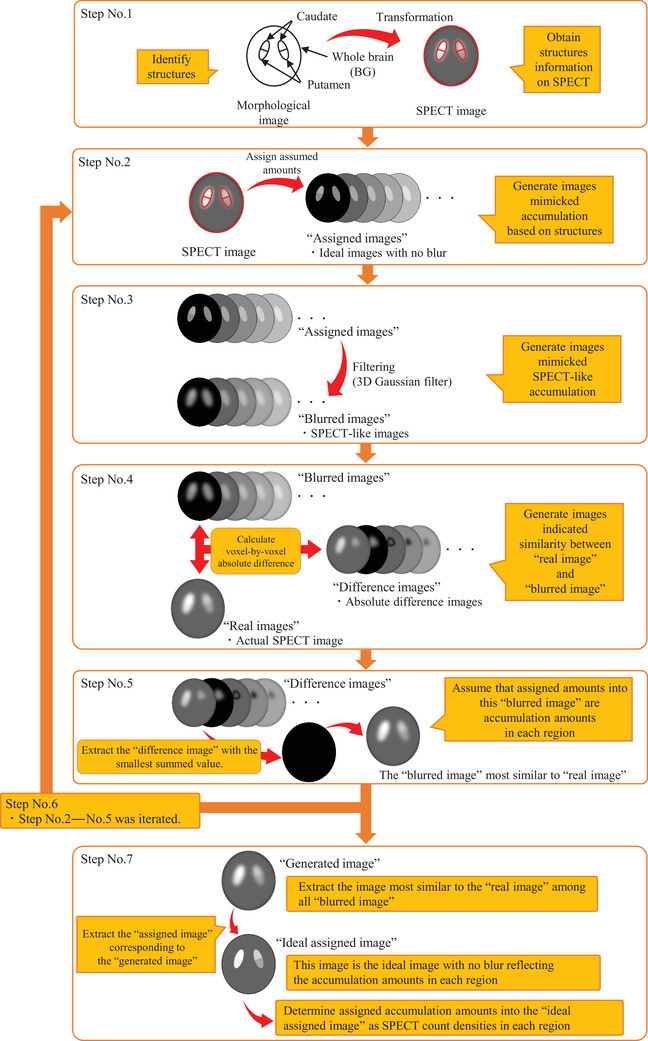
Flow of the calculation process by the proposed method. The proposed method consists of seven steps. This figure shows the flow from Step (1)–(7) of the proposed method. The series from Steps 2–5 was performed 10 times to compute more accurate accumulation amounts

The proposed method is a region‐based method that seeks to identify the amount of accumulation for each region by evaluating the image approximated to the SPECT image. The flow of the calculation process by the proposed method is shown in Figure 2.

**FIGURE 3:**
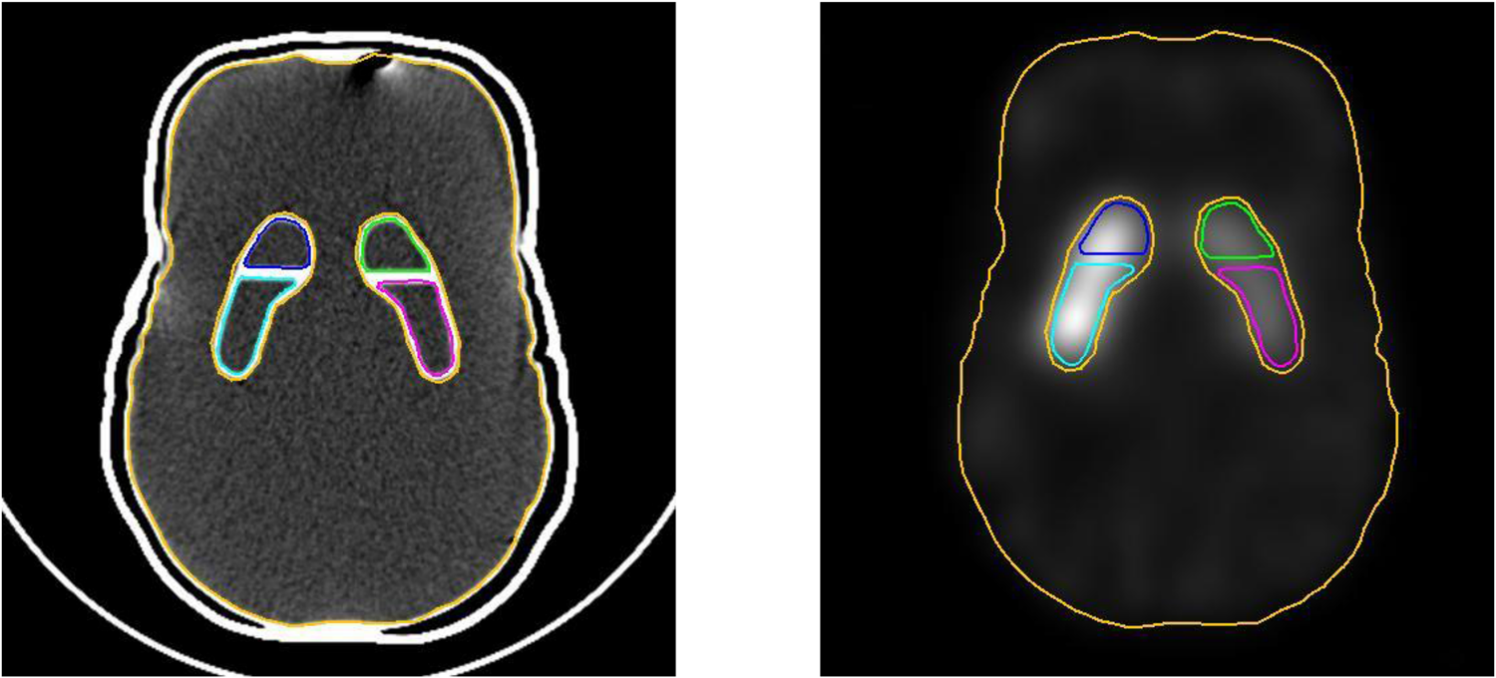
The establishment of the volume of interests (VOIs). The left image shows the establishment of VOIs of the caudate, putamen, and the whole brain as background (BG) regions on computed tomography. The right image shows the VOIs of each region on single‐photon emission computed tomography

Step (1): Using the PMOD software version 3.903 (PMOD Technologies LCC, Zurich, Switzerland), we manually established the VOIs of the left and right caudate/putamen regions and the VOI of the whole brain (including the striatum) for the BG region on the morphological images (CT images was used in this study). Using PMOD software, VOIs were applied to the SPECT image in agreement with the coordinates of the VOIs on the CT image (Figure 3). However, the shape of the VOIs changed based on the voxel size. The positional information of the left and right caudate/putamen and BG regions on SPECT images were obtained from each VOI.

Steps (2)–(7) were processed on our program built using Python version 3.7.3.

Step (2): The assumed accumulation amounts were assigned to each region based on the positional information of the left and right caudate/putamen and BG regions obtained by Step (1). By this step, ideal images without blur were generated. We defined these images as “assigned images.”

Step (3): The SPECT spatial resolution in clinical conditions (FWHM: *x* = 11.52 mm, *y* = 11.89 mm, *z* = 11.71 mm) was measured using a line source phantom (SPECT phantom JSP, Kyoto Kagaku Co., Ltd., Kyoto, Japan). In this study, the spatial resolution was evaluated using a line source phantom following the national standard in Japan.^19^ Therefore, the *z*‐direction FWHM could not be measured. Since the spatial resolution of SPECT is considered not to vary in the *x*‐, *y*‐, and *z*‐directions, the mean value of the *x*‐direction and *y*‐direction FWHM was used for the *z*‐direction FWHM. A three‐dimensional (3D) Gaussian filter equivalent to this spatial resolution was applied to the “assigned images” from Step (2). The SPECT‐like spatial resolution images were generated. We defined these images as “blurred images.”

Step (4): We defined the image, obtained by the actual measured SPECT and normalized with the maximum value, as the “real image.” The voxel‐by‐voxel absolute differences between the “blurred images” generated in Step (3) and “real image” were calculated, thereby generating absolute difference images between the “real image” and the “blurred images,” defined as “difference images.”

Step (5): The summed value of “difference image” represents the degree of similarity between “blurred image” and “real image.” Therefore, the “difference image” with the minimum summed value was extracted. We defined the assumed accumulation amounts assigned to the “blurred image” used when the extracted “difference image” was created as the accumulation amounts in the left and right caudate/putamen and BG regions.

Step (6): The assumed accumulation amounts assigned to each region in Step (2) were first selected as the approximate amounts. As Steps (2)–(5) was iterated, the assumed accumulation amounts were updated in detail. Through the iterations, we scrutinized for more accurate accumulation amounts. Details of updating process are given in the Supporting information.

Step (7): After Steps No. 2–No. 5 was iterated, the final “blurred image,” which was the closest to the “real image,” was extracted. We subsequently defined this final extracted “blurred image” as the “generated image.” The “assigned image” corresponded to the “generated image” was extracted. This “assigned image” was the ideal image with no blur reflecting the accumulation amounts in the left and right caudate/putamen and the BG regions, and we defined this “assigned image” as the “ideal assigned image.” We finally determined the assumed accumulation amounts of the left and right caudate/putamen and BG regions assigned to the “ideal assigned image” as SPECT count densities for each region. The SPECT count density obtained by the proposed method was defined as *C*_proposed_.We computed the SBR and caudate‐putamen ratio (CPR) with the Equation (1) and (2) respectively.

(1) $$\mathrm{SBR}=\frac{C_{\mathrm{proposed}\;}\left(\mathrm{caudate}\;\mathrm{or}\;\mathrm{putamen}\right)-C_{\mathrm{proposed}}\;(\mathrm{BG})}{C_{\mathrm{proposed}}\;(\mathrm{BG})}$$

(2) $$\mathrm{CPR}=\frac{C_{\mathrm{proposed}\;}\;(\mathrm{caudate})-C_{\mathrm{proposed}}\;(\mathrm{BG})}{C_{\mathrm{proposed}}\;(\mathrm{putamen})-C_{\mathrm{proposed}}\;(\mathrm{BG})}$$

### 2.4 Comparison method

#### 2.4.1 CT‐guide method

VOIs, as shown in Figure 3, were used (the striatal region was not included in the BG). We computed the SPECT count densities for the left and right caudate/putamen and BG regions using these VOIs. The SPECT count density computed by the CT‐guide method was defined as *C*_CT‐guide_. The *C*_CT‐guide_ did not consider PVE.

#### 2.4.2 Conventional PVC method

We used two conventional methods of PVC, the GTM method and the RBV method. The GTM method is a VOI‐based PVC method described by Rousset et al.,^10^ while the RBV method, described by Thomas et al.,^11^ is a voxel‐wise PVC that extends the GTM method. We used the same FWHM as in Section 2.3 and the same VOIs as mentioned in Section 2.4.1 (Figure 3). The SPECT count density computed by the GTM and RBV methods were defined as *C*_GTM_ and *C*_RBV_, respectively.

#### 2.4.3 Southampton method

The Southampton method, described by Tossici‐Bolt et al.,^12^ is widely used in Japan and uses a large VOI, including the entire striatum, to correct the PVE. The SPECT count density computed by this method was defined as *C*_Southampton_. We used the software DaTView (AZE, Tokyo, Japan), which adopted the Southampton method to compute the *C*_Southampton_ for the left and right striatum and BG regions.

### 2.5 Statistical analysis

We measured the actual count density (counts/s g) of ^123^I solution filled into the phantom by an auto‐well counter. We evaluated the Pearson's correlation coefficients (CoC) between the actual count density and the SPECT count densities computed by five methods (i.e., *C*_proposed_, *C*_CT‐guide_, *C*_GTM_, *C*_RBV_, and *C*_Southampton_). The significance of the differences between the CoCs of four methods, excluding the Southampton method, were tested using the Meng–Rosenthal–Rubin method^20^ with Bonferroni correction for multiple comparisons. The difference between CoCs could not be tested in the Southampton method because the VOIs used in the analysis of the Southampton method were different from the ones used in other methods; *p*‐values < 0.05 were considered statistically significant.

In Phantom 2, SBR and CPR (calculated by the actual count density of ^123^I solution in the phantom) were defined as theoretical values. The absolute errors between the SBR calculated by the proposed method and the theoretical value were calculated using the Equation (3) (the absolute errors for CPR were also calculated):

(3) $$\begin{array}{ccc} & & \mathrm{Absolute}\;\mathrm{error}=\mathrm{calculated}\;\mathrm{value}\\ & & \;\left(\mathrm{the}\;\mathrm{proposed}\;\mathrm{method}\right)-\mathrm{theoretical}\;\mathrm{value}. \end{array}$$

## 3 RESULTS

**FIGURE 4:**
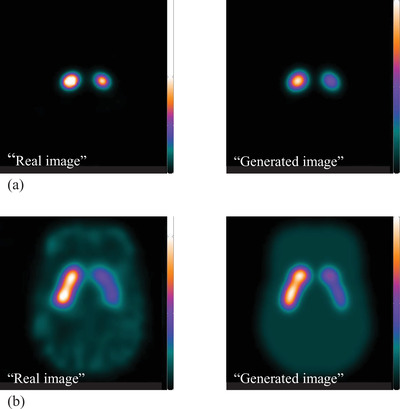
Comparison between the “real image” and the “generated image”. The “real image” and the “generated image” obtained from Phantom 1 (a) and the image obtained by Phantom 2 (b)

The comparison between the “real image,” which is the actual measured SPECT image and the “generated image” obtained by the proposed method is represented in Figure 4. The images shown in Figure 4 had the same display conditions (color range and slice position), and the counts and contrasts of the caudate, putamen, and BG in the “generated image” were visually similar to them in the “real image.”

**FIGURE 5:**
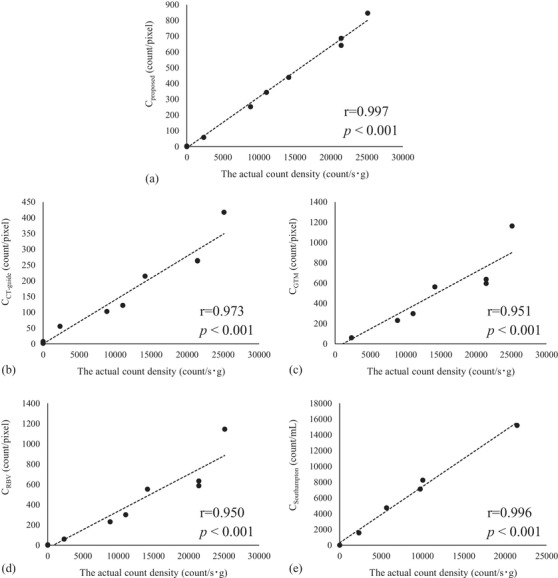
The correlation between the actual count density and the single‐photon emission computed tomography (SPECT) count density. The figure shows the correlation between the actual count density of the ^123^I solution filled in the phantom and the SPECT count densities computed by (a) the proposed method, (b) the computed tomography guide method, (c) the geometric transfer matrix method, (d) the region‐based voxel‐wise method, and (e) the Southampton method

The CoC between the actual count density filled into the phantom and the SPECT count densities calculated by five methods are shown in Table 2. The CoC between the actual count density filled into the phantom and the *C*_proposed_ (CoC_proposed_), *C*_CT‐guide_ (CoC_CT‐guide_), *C*_GTM_ (CoC_GTM_), *C*_RBV_ (CoC_RBV_), and *C*_Southampton_ (CoC_Southampton_) were 0.997, 0.973, 0.951, 0.950, and 0.996, respectively (Figure 5; all CoCs were significant [*p* <0.001]). Upon testing the significant difference of CoCs, CoC_proposed_ was significantly higher than the CoCs of the other methods (except the Southampton method). No significant difference was found between other methods. Although the significant difference between CoC_proposed_ and CoC_Southampton_ could not be tested as mentioned in Section 2, CoC_proposed_ was almost comparable to CoC_Southampton_.

**TABLE 2:** The correlation coefficients between the actual count density and five methods

			**The difference test of correlation coefficient *p*‐value** *		
	**Correlation coefficient**	***p*‐Value**	**Proposed method**	**CT‐guide method**	**GTM method**
Proposed method	0.997	<0.001	–	–	–
CT‐guide method	0.973	<0.001	<0.001	–	–
GTM method	0.951	<0.001	<0.001	0.042	–
RBV method	0.950	<0.001	<0.001	0.042	0.684
Southampton method	0.996	<0.001	–	–	–

* Correlation is significant at the 0.008 level with Bonferroni correction.

In Phantom 2, the errors between the SBR calculated by the proposed method and the theoretical SBR, calculated by the actual count density of ^123^I solution filled into the phantom, are shown in Table 3. The SBR calculated by the proposed method was overestimated in all regions. In addition, the absolute errors between the CPR calculated by the proposed method and the theoretical CPR were approximately 0.1, and the CPRs calculated by the proposed method approached the theoretical CPRs (Table 4).

**TABLE 3:** Comparison between the theoretical specific binding ratio (SBR) and SBR obtained by the proposed method

	**Right caudate**	**Right putamen**	**Left caudate**	**Left putamen**
Theoretical SBR	9.17	9.17	4.73	3.79
Calculated SBR	11.83	11.07	5.92	4.36
Absolute error	2.65	1.89	1.19	0.57

*Note*: Theoretical SBR, the SBR calculated by the actual count density of ^123^I solution filled in the phantom. Calculated SBR, the SBR calculated by the proposed method. Absolute error, the difference between the theoretical SBR and calculated SBR.

**TABLE 4:** Comparison between the theoretical caudate–putamen ratio (CPR) and CPR obtained by the proposed method

** **	**Right striatum**	**Left striatum**
Theoretical CPR	1.00	1.25
Calculated CPR	1.07	1.36
Absolute error	0.07	0.11

*Note*: Theoretical CPR, the CPR calculated by the actual count density of ^123^I solution filled in the phantom. Calculated CPR, the CPR calculated by the proposed method. Absolute error, the difference between the theoretical CPR and the calculated CPR.

## 4 DISCUSSION

It is desirable to evaluate the accumulation amounts in the caudate and putamen separately, considering the pathology of PD. However, it is difficult to evaluate the accumulation amounts due to PVE accurately. Therefore, this study aimed to verify whether the newly proposed method could accurately evaluate the accumulation amounts in the caudate and putamen with reduced PVE and compared the proposed method with the conventional PVC methods.

The SPECT count densities computed by all methods used in this study significantly correlated with the actual count density filled in the phantom (Figure 5, Table 2). The CoC_proposed_ was significantly higher than the CoCs of the other methods (except the Southampton method, Table 2). The differences between the CoCs of the other methods (except the Southampton method) were not significant (Table 2). Although the differences between the CoC_GTM_/CoC_RBV_(the conventional PVC method) and the CoC_CT‐guide_ (no PVC method) were not significant, the CoC_GTM_ and CoC_RBV_ tended to be slightly lower than that of the CoC_CT‐guide_ (Table 2). In this study, we used a line source phantom to measure the FWHM used in the PVC methods. A previous study had reported that the calculation using physical FWHM might bring false results since the physical FWHM measured using a line source phantom is different from the FWHM measured in the acquisition condition of the striatal setup.^21^ This is one reason the CoC_GTM_ and CoC_RBV_ were lower than the CoCs of the other methods. Moreover, since the GTM method and RBV method do not compare with the actual measured image and value in the calculation process, the error between the processing results and the actual image/value was large. Contrarily, because the proposed method examined the most probable accumulation amounts while repeatedly comparing with the actual measured image, the proposed method could compute the amounts more accurately. Moreover, the proposed method worked well using the same physical FWHM used in the GTM method and the RBV method; therefore, the difference between the calculation processes described above contributed to the influence of the FWHM on calculated results.

Among the various PVC methods, it has been reported that the RBV method brought better results than others.^11^ In this study, the difference between the CoC_GTM_ (the region‐based method) and CoC_RBV_ (the voxel‐based method) was not significant (Table 2). Because it was a phantom study with uniform activity distributions, the difference between GTM and RBV methods was not considered significant. It has been reported that the voxel‐based PVC methods were not noise‐robust,^22^ and there were cases with artifacts.^23^ The half‐life of the ^123^I tracer is longer than the PET tracer or ^99m^Tc tracer and cannot increase the dosage from the perspective of exposure. Therefore, the obtained image used ^123^I tracer is noisier than the PET image or ^99m^Tc image. Since the proposed method was region‐based PVC, which balanced the accumulation amounts in each region and performed processing that brought it closer to the actual measured image, examining true count density suppressing the effects of noise, the highest correlation was obtained in the proposed method. The region‐based PVC computes the average value in the region, suppressing the influence of noise; contrarily, the voxel‐based PVC may emphasize noise. However, the voxel‐based PVC can consider the non‐uniformity accumulation and be useful when the evaluation is combined with visual interpretation.^21^ By applying a process that treats several to several tens of voxels as a group and performs PVC, which might be performed by taking respective advantages of region‐based and voxel‐based PVC.

It is expected that the Southampton method can relatively suppress noise since a large VOI used in the Southampton method included more counts than the other methods used in this study, which analyses the caudate and putamen separately. As expected, the *C*_Southampton_ strongly correlated with the actual count density (correlation of 0.996). The significant difference test between CoC_proposed_ and CoC_Southampton_ could not be performed due to the difference in the analysis process. However, CoC_proposed_ was comparable to CoC_Southampton_. In other words, the proposed method could analyze the caudate and putamen separately, resulting in the comparable accuracy of the Southampton method. However, the SBRs were overestimated in all regions (Table 3). This overestimation was because the contrast between the striatum and BG was overemphasized due to over‐correction by SC, AC, and PVC. It has been reported that SC and AC improve the quantitative evaluation compared to no corrections.^6^, ^7^, ^14^, ^24^ In addition to SC and AC, PVC also has been reported to be valuable in quantitative evaluation.^6^, ^7^ Although the SBR is overestimated by the over‐correction, these SBRs did not indicate the absolute theoretical SBRs calculated by the actual count density of ^123^I solution filled into the phantom. Since *C*_proposed_ strongly correlated with the actual count density filled in the phantom, the SBRs calculated by the proposed method did not represent the absolute theoretical SBR but the relative theoretical SBR on SPECT image. In support of this, the CPRs were extremely close to the absolute theoretical CPRs (Table 4). The *C*_proposed_s of the caudate and putamen were increased by the corrections; however, the caudate was divided by the putamen in the CPR, which indicates the influence of the over‐correction was canceled. Therefore, the CPRs approached the absolute theoretical CPRs. In PD, the accumulation begins to decrease from the putamen.^16^ Hence, while evaluating the accurate CPR, the proposed method can detect the accumulation reduction in the putamen with high sensitivity, which is expected to be beneficial in clinical practice.

Although the proposed method is similar to the BasGan^25^ or Van Cittert (VC) method,^26^ differences exist. The proposed method uses morphological images. In the proposed method, the accumulation amounts in each region are narrowed down from a wide range, and processing is performed to gradually hit the true amounts. Moreover, since the analysis is performed by comparing many image patterns, it is different from the other two methods. Therefore, though it takes more time than the other methods, there is a possibility that more accurate values may be calculated.

This study had certain limitations. Only phantoms were examined in this study. The image, approximated most to the actual measured SPECT image, was examined by generating tens of thousands of SPECT‐like images. As a result, the process took time, which is not suitable for clinical practice in the present form. Moreover, our examination was region‐based for the proposed method, which can be extended to a voxel‐based method. In future, it is necessary to compare the results of this study with a voxel‐based method extending the proposed method. Although some improvements are needed in the proposed method, we obtained highly accurate results. Issues to be examined in future include shortening of processing time, voxel‐based comparative study, application to clinical cases, and comparing the proposed method with the conventional methods in clinical cases. In clinical use, CT images cannot distinguish between the caudate and putamen; therefore, it would be better to use MR images. Moreover, adding the cerebral ventricles (low or no accumulation) regions to the calculation process in addition to the caudate, putamen, and BG regions will allow more accurate calculations.

## 5 CONCLUSION

In conclusion, using the newly proposed method, the accumulation amounts in the caudate and the putamen considering PVE could be calculated separately. We proved the validity of the proposed method by comparing it with conventional PVC methods. However, this study was conducted using phantoms. Therefore, further research in clinical settings is warranted.

## Supporting information

The details of updating process in the proposed method

Based on the positional information of the left and right caudate/putamen BG regions obtained by Step (1), five values (0, 0.5, 1.0, 1.5, and 2.0) were assumed to be the accumulation amounts and were assigned to each region in the first step of Step (2). Ideal images with no blur (“assigned image”) were generated with this step. Since five assumed accumulation amounts (0, 0.5, 1.0, 1.5, 2.0) were assigned to the five regions (the left and right caudate/putamen and BG regions), 5^5^ = 3125 “assigned images” were generated. In Step (3), these “assigned images” were blurred by applying a 3D Gaussian filter, which generated the same number of “blurred images” as the “assigned images.” Then, the “blurred image” which was most approximated to the “real image” among these “blurred images” was extracted, and the accumulation amounts among the five regions were determined. This is the first process in Step (1)–(5). These accumulation amounts obtained in the first process were from the wide range of assumed accumulation amounts, 0, 0.5, 1.0, 1.5, and 2.0 (the range of the assumed accumulation amounts was 0–2.0, and the interval of the assumed accumulation amounts was 0.5). Therefore, the assumed accumulation amounts were needed to update in detail to examine the “blurred image” that was more approximated to the “real image.” The range and interval of the assumed accumulation amounts assigned in the next process were updated to half of the range and the interval of the assumed accumulation amounts assigned in the pre‐process of Step (2).

For example, it hypothesizes that the “blurred image” is most approximated to the “real image” when 1.0 as the assumed accumulation amount is assigned to a certain region. Among the assumed accumulation amounts (0, 0.5, 1.0, 1.5, 2.0) assigned in the first process, the values before and after the 1.0, which is the value when the “blurred image” is most approximated to the “real image” are determined as the range of the assumed accumulation amounts assigned in the second process. In other words, the range of the assumed accumulation amounts assigned in the second process is 0.5–1.5. Moreover, although the interval of the assumed accumulation amounts of 0, 0.5, 1.0, 1.5, and 2.0 is 0.5 in the first process, the interval is 0.25 in the second process, which is half of the first process. Therefore, the five values, 0.50, 0.75, 1.00, 1.25, and 1.50, are reassigned to the same region as the assumed accumulation amounts in the second process. An exception process was performed in case the “blurred image” was most approximated to the “real image” when the assumed accumulation amount was zero since the process in the proposed method was performed so that the assumed accumulation amount was never negative.

In case the “blurred image” is approximated most to the “real image” when the assumed accumulation amount is 0, the range of the assumed accumulation amount assigned in the second process is not the value before and after 0, which is assigned in the first process, and as an exception, only the value after the 0 is used. In other words, the range of the assumed accumulation amounts assigned in the second process will be from 0 to 0.50 in this update. However, the interval of the assumed accumulation amounts is 0.25, as with the usual process. Therefore, three values, 0, 0.25, and 0.50, as the assumed accumulation amounts, are assigned again in the second process in case the “blurred image” is most approximated to the “real image” when the assumed accumulation amount is 0. This update process of the assumed accumulation amounts was performed in all regions (the left and right caudate/putamen and BG regions). Subsequently, Steps (2)–(5) was iterated to approach the more probable values. This series (Steps (2)–(5)) was performed 10 times.

We also investigated whether it was reasonable to perform Steps (2)–(5) 10 times in the present study. The variability rate of SBR with the number of processes was calculated using the following formula:

(4) $$\mathrm{Variability}\;\mathrm{rate}\;\;\left(\%\right)=\frac{\mathrm{SB}R_{n+1}-\mathrm{SB}R_{n}}{\mathrm{SB}R_{n}}\times 100$$

**FIGURE 6:**
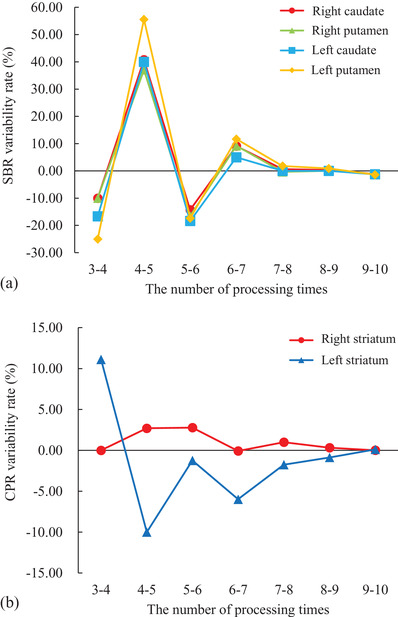
The relationship between the number of processing times and the variability rate. (a) Shows the relation between the number of processing times and the variability of specific binding ratio (SBR). SBRs were converged as the processing was repeated. When the number of processing times was more than seven, the variability rates of the SBR were less than 5%. (b) Shows the relation between the number of processing times and the caudate/putamen ratio variability. This relation showed a similar trend to the SBR

SBR*_n_* represents the SBR in the *n*th process. The variability rate of CPR was also calculated using Equation (4). The variability rate of SBR and CPR with the number of processes is shown in Figure 6. We considered SBR and CPR to converge when the number of processes was over seven since SBR and CPR variability rates were less than 5%. Therefore, 10 times, which was the number of processes used in this study, was sufficient to converge.

## AUTHORs’ CONTRIBUTION

Katsuhiko Kato is the guarantor of integrity of the entire study. Yoshinori Ito, Naotoshi Fujita, and Katsuhiko Kato contributed to the conception and design of the study. Yoshinori Ito, Naotoshi Fujita, Tomohiro Tada, Shinji Abe, Shinji Naganawa, and Katsuhiko Kato contributed to the acquisition of data. Yoshinori Ito contributed to the analysis of data. Yoshinori Ito, Naotoshi Fujita, and Katsuhiko Kato contributed to the manuscription preparation. Naotoshi Fujita, Kazuhiro Hara, Masahisa Katsuno, Shinji Abe, Shinji Naganawa, and Katsuhiko Kato contributed to the manuscript editing. All authors read and approved the final manuscript.

## CONFLICT OF INTEREST

The authors declare that there is no conflict of interest that could be perceived as prejudicing the impartiality of the research reported.

## Data Availability

The data that support the findings of this study are available from the corresponding author upon reasonable request.
